# Contrasting Birth Preferences to Practices in El Paso, Texas

**DOI:** 10.3389/fgwh.2022.830512

**Published:** 2022-03-29

**Authors:** Rachel S. Curtis, Regina Vadney, Carina Heckert, Cathy Román

**Affiliations:** ^1^Independent Researcher, El Paso, TX, United States; ^2^Department of Sociology and Anthropology, The University of Texas at El Paso, El Paso, TX, United States

**Keywords:** midwifery, informed consent, childbirth, maternity care, El Paso, birth center, home birth, patient autonomy

## Abstract

Despite calls for increased access to midwifery and a reduction in unnecessary labor interventions by the World Health Organization, the American College of Obstetrics and Gynecologists, and the American Public Health Association, for many birthing parents in the United States, this model remains out of reach. Only 10% of U.S. births are attended by midwives, and in Texas, which leads the nation in maternal morbidity and mortality, that number is <7%. This study examines an unmet demand for personalized, low-intervention midwifery care in El Paso, Texas and the surrounding area through surveys and focus groups aimed at exploring women's perceptions of their birthing experiences and access to different models of perinatal care. Resulting data suggests a high level of satisfaction with midwifery among those who were able to access it, while those who had used obstetric care often reported limited options and feelings of trauma.

## Introduction

Childbirth is a normal, healthy physiological function of all mammals. Midwives are birth attendants trained to recognize, protect, and support this normal process. Limiting interventions or intrusive management of labor unless medically indicated is a hallmark of midwifery care. For patients who develop conditions during pregnancy or labor that are outside of healthy parameters, obstetric specialists are consulted for concurrent care or a complete transfer of care.

With elevated maternal mortality rates in the United States (1), birth practices have come under increasing scrutiny in the last decade. It has become broadly recognized that common obstetric practices are of limited or uncertain benefit, and the American College of Obstetricians and Gynecologists (ACOG) has issued statements calling for limiting interventions (2). ACOG has published numerous commentaries, reports, and studies supporting collaborative practice models to integrate midwifery care with obstetrics in order to achieve this goal and improve maternal healthcare (3–6).

Beyond clinical outcomes, many birthing people desire a more patient-centered and physiological birth experience than what is often available. The United Nations recognizes the importance of patient-centered care in a 2018 guideline, stating, “The UN Global Strategy for Women's, Children's and Adolescents' Health seeks to ensure that women give birth in an environment that in addition to being safe from a medical perspective, also allows them to have a sense of control through involvement in decision making and which leaves them with a sense of personal achievement (7)”.

The present study examined women of childbearing age seeking a low-intervention, physiological birth experience through a survey (*n* = 324) and two focus groups with participants who had previously completed the survey. While the survey provided primarily quantitative data, we also included several open-ended questions. Themes that emerged in the open-ended questions were explored in greater depth through the focus groups. Quantitative data revealed stark differences in participants' birth preferences to their actual experiences. We analyzed qualitative data to further explore feelings about birth experiences and quality of care, giving attention to differences in experiences based on the type of provider attending the birth. We also explored participants' knowledge of and desire for midwifery care.

Salient themes included limited options, comparisons between midwifery and obstetric practices, comparisons between hospital and out-of-hospital birth experiences, and the appeal of midwifery care.

## Materials and Methods

### Research Context

In 2019, birthing families in El Paso, Texas had the following birthing locations available: five private hospitals (no midwives), one county hospital with certified nurse midwives (CNMs), one military hospital with CNMs, three free standing birth centers with certified professional midwives (CPMs), and home birth. The hospitals with CNMs were not able to guarantee patients a midwife during delivery, even if they had gotten their prenatal care with a midwife because midwives were not staffed 24/7 in either labor and delivery unit.

Because health insurances in this area cover services provided by CNMs but not CPMs, and because CNMs in this area only see patients in hospitals, families in El Paso could only use their insurance to pay for births in a hospital. The one exception was a free-standing birth center that obtained coverage for a brief period by federal military insurance, increasing access to this option for families stationed at a major military base in the city.

Moreover, CPMs do not have privileges to work in hospitals. Therefore, when transfers to hospitals occur, or if clients become too high risk for midwifery care during their pregnancy, they lose access to their midwives and encounter a new set of providers.

### Community Needs Survey

We collected 324 responses from women of childbearing age (15–49 years old) from August to December 2019 in the El Paso, Texas and southern New Mexico region. The survey was designed to gauge interest in a free-standing birth center staffed by CNMs with access to the hospital with the same provider, all of which could be billed to insurance. At the time of the survey, families had access to either high-volume hospitals almost exclusively attended by obstetricians, or to birth out of hospital with midwives unable to accompany them in event of a transfer to hospital. Thus, one aim of the survey was to evaluate to what extent there was an unmet need within the community for a greater array of midwifery options.

Given that the goals of the study were to demonstrate demand for a particular model of care, rather than to draw conclusions about the population at large, a convenience sample was determined to be most appropriate. We wanted to take a closer look at the needs and experiences of the subset of the birthing population for whom midwifery care may carry the greatest import. Considering this aim, we recruited participants through groups and organizations that are known locally to serve women with an interest in low-intervention childbirth.

The survey was shared at breastfeeding events, to contact lists of local doulas and midwives, and on various Facebook groups for mothers, breastfeeding support, and women interested in natural childbirth. The survey was not intended to capture a representative sample of the population, but to demonstrate a need for expanded maternity care options.

The survey consisted of four sections: Ideal Birth Scenario, Hospital Birth Experience, Out-of-Hospital Birth Experience, and Demographic information. Women who had not yet had a child filled out only the first and last sections. There were a total of 29 questions, including the option to add comments at the end of each section. The closed-ended questions rated preferences on a likert scale (e.g., “very important” “somewhat important” “not important”), while others included a list (e.g., “which of these options for pain relief were offered to you”).

Demographic information included age, ethnicity, level of education, income, type of health insurance, and zip code.

A total of 19 responses were excluded either for being outside the defined age range, being unable to confirm a delivery in the defined geographic area, and duplicate responses. Results were tabulated from 305 completed surveys.

### Focus Groups

Many survey participants added information in the open spaces provided at the end of each section of the survey. Some of these contributions were impassioned and intriguing, and we wanted to hear more of what they had to say. We conducted focus groups to amplify these voices and explore the reasons behind their birth preferences. The focus groups were semi-structured, meaning that the research team developed a set of talking points in advance, but we maintained flexibility with the questions to encourage open-ended conversation within the groups.

Invitations to partake in the focus groups were emailed to 41 of the survey respondents who answered open-ended questions in the survey. A total of 10 women attended two focus groups held in November 2019, the first with seven participants and the second with three. Questions centered around perceptions of midwifery, personal feelings about birth as a unique experience, how birth locations and providers impacted care, and thoughts about the options currently available. The conversations were recorded and transcribed.

Questions were standardized beforehand and qualified for exemption from the Internal Review Board of the University of Texas at El Paso; each participant was given a Study Information Sheet.

### Data Analysis

We used SPSS software to analyze the survey data to produce summary statistics and cross tabulations. We analyzed the focus group transcriptions and open-ended questions from the survey using Atlas.ti qualitative data analysis software. Inductive reasoning guided our analysis, in that we coded the data line-by-line in relation to themes that emerged.

## Results

Our data revealed several significant dichotomies: preferences vs. options, common midwifery practices vs. common obstetric practices, and satisfaction with birth experiences in hospital vs. out of hospital. Moreover, it was clear that women were deeply impacted by their birth experiences, positively or negatively, even years later.

Survey respondents fell into three categories: those who birthed in hospital with an obstetrician (the largest group), those who birthed out of hospital with midwives (CPMs), and those who birthed in hospital with CNMs (the smallest group). In the majority of cases, patients referring to a hospital birth received care from an obstetrician.

### Preferences vs. Options

Over a range of choices, a contrast emerged between what women would like in labor and what they had in labor. A few key findings from the quantitative data are outlined here.

#### Freedom of Movement

**Figure 1:**
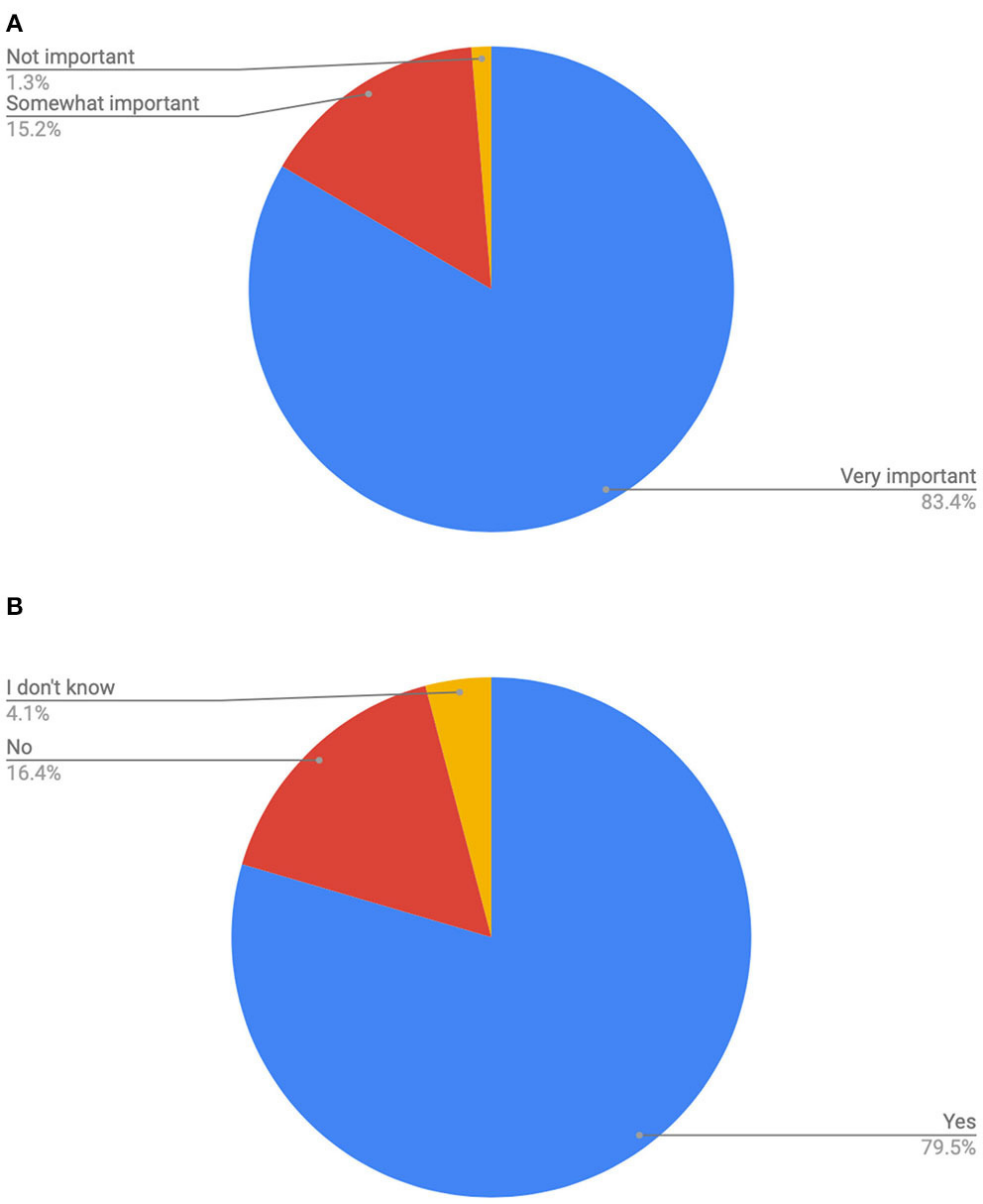
**(A)** Count of how important is it that you are free to move as you like in labor? (Get in a tub or shower, go outside, change position…). **(B)** Count of were you restricted to bed at any time in labor?

Overwhelmingly, pregnant people wanted to move freely when they were in labor, with only 1.3% stating this was not important. Among respondents who birthed in hospital, nearly 80% were restricted to bed at some point. Similarly, 78% said they were not given time with the monitors off their bellies so they could move more freely (see Figures 1A,B).

#### Eating and Drinking

This divide was also seen on the question of restricting food and drink; while only 8% of those surveyed said they did not think this was important, 78% said that they were prevented from eating or drinking.

#### Pain Relief

Only 1.7% of respondents felt it was not important to have options for pain relief other than intravenous medications or an epidural. While many did have position changes or other non-pharmacological methods offered at hospitals, this was in less than a third of all cases. In contrast, epidurals and IV medications were offered to 86% and 50% of participants, respectively.

#### Involvement in Decision Making

**Figure 2:**
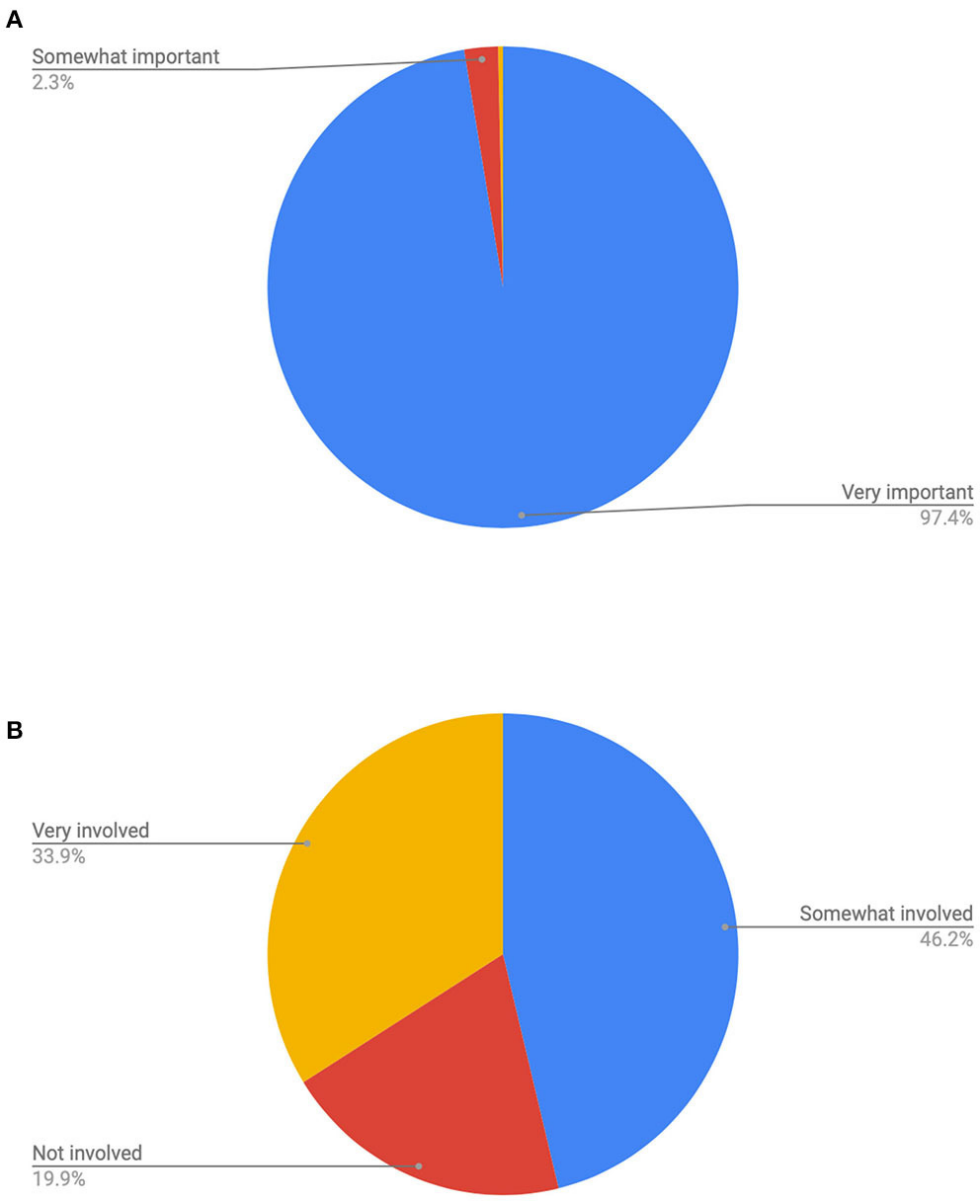
**(A)** Count of how important is it to feel that you are included in making decisions regarding your care during labor? **(B)** Count of how much were you involved in making decisions regarding your care during labor.

This was the most unanimous preference expressed in the survey, with a full 97.4% ranking involvement in decision making as very important, and another 2.3% saying it was somewhat important. Only 0.3% did not give this any importance. In the hospital setting, only 34% of respondents felt they were very involved in decisions regarding their care. One in five, or 20%, did not feel they were involved in decision making (see Figures 2A,B).

### Common Midwifery Practices vs. Common Obstetric Practices

Comparing women's preferences to the options they found available underscores many practices that have become standardized in hospitals and obstetric care. Among the majority of our respondents who birthed with obstetricians, restricting oral nourishment, pharmacological management of pain, and hierarchical decision making that centered the provider's and facility's needs over the patient's, were routine.

We asked respondents who birthed at home or in a birth center with midwives the reason for that decision. They frequently cited a desire to avoid common obstetric practices, often accompanied by concerns that they would be pressured into procedures. This mother's response echoed that of many others:

I wanted no unnecessary interventions, I wanted to not have to fight during labor to be an active decision maker in what happened to me and my baby, I wanted to be surrounded by people who appreciate and understand physiological labor and how important it is to be hands off unless there's a problem, and I wanted to be able to eat and drink and move around and use the bathroom as I wanted and not be told I'm not allowed to.

In sum, families who chose midwifery care were seeking a hands-off, low-intervention environment that would allow them to remain in control of decisions (see Table 1).

**Table 1:** Importance of the midwifery model of care (*n* = 304).

**Questions related to common midwifery practices**	**Not important**	**Somewhat important**	**Very important**
How important is it that the person who delivers your baby is the person who has provided your prenatal care?	2.3%	17.8%	79.9%
How important is it that you are free to move as you like in labor? (Get in a tub or shower, go outside, change positions freely, not be on your back to push.)	1.3%	15.5%	83.2%
How important is it to have options for pain management other than medication through an IV or an epidural? (nitrous oxide, doula, aromatherapy, tub, massage, etc.)	1.6%	15.1%	83.2%
How important is it to be able to eat and drink during labor?	7.9%	33.9%	58.2%
How important is it to have the same people care for you the entire time during labor, without shift changes?	6.6%	31.6%	61.8%
How important is it to feel that you are included in making decisions regarding your care during labor?	0.3%	2.3%	97.4%
How important is it that your insurance cover the cost of your birth?	2.6%	12.8%	84.5%

### Dissatifaction With Hospital Births

When discussing experiences birthing in hospital, respondents frequently named a lack of autonomy, violations of consent, and coercion. The inability to refuse Pitocin (a medication to induce or augment uterine contractions) arose repeatedly.

#### Lack of Autonomy

Respondents felt their choices were restricted at the hospital, with things that were important to them being disregarded. Many discussed not being allowed to do what they wanted, or being obligated to do things they did not want to do. Themes of negligence and lack of attention also surfaced. One mother recalled a general sense of impotence, “I had two births in a hospital, and would never want to give birth there again. You have no say in anything”.

Another spoke more specifically about the ways she was disempowered during a birth in 2017:

From the time my water broke until I gave birth to my son, 31 h later, I was restricted to the bed. They were unable to find a doctor to deliver me for 6 h. Unable to eat, unable to move, the only thing I was offered was a big bouncy ball to put between my knees. I would never consider giving birth there again.

#### Informed Consent

In addition to feeling they were not included in decision making, mothers specifically named violations of consent and having procedures performed without their permission. This mother's sense of being wronged remained raw, several years later, “I was given Pitocin IN MY SLEEP without my consent … I was also given an episiotomy without consent. I was treated like a child at 30 years old and talked down to constantly. I was given zero control.”

An experienced mother who had birthed children at home, in a birth center, and in hospital discussed disrespect and violations of consent with her last baby.

We had a home birth that ended in a hospital transfer. At home I was allowed to do as I pleased. At the hospital I had no rights it seemed. I was treated horribly if I questioned anything or declined anything. Procedures were done without my permission and it was my worst birth experience. This was my 5th child.

#### Coercion

Respondents reported a loss of control over the birthing process and their bodies, and care providers who pressured them into procedures they did not want. This happened both with nurses and with physicians, and women's feelings about how they were treated remained powerful. One recalled, “Before my OB arrived the nurses were trying to force me to get an epidural, until I explained to them I would call my doctor. Then they stopped trying to make me get it.”

Another said simply, “It was downright abusive and horrible.”

While many participants expressed misgivings with their hospital birth experiences, some prepared and advocated for greater choices during their hospital deliveries, including seeking midwifery care in hospital and hiring doulas. These mothers credited the involvement of midwives and doulas with more satisfying births. One mother of two told us, “First thing I did was hire a doula and educated myself on the birthing process. It's unfortunate hospitals only offer epidurals for pain. Luckily, my doula and I had other options prepared.”

### Satisfaction With Birth Center and Home Birth

We asked mothers why they chose not to birth in the hospital, even though in many cases this meant paying out of pocket for all their medical care. They named many routine obstetric procedures they wanted to avoid, such the restriction of movement or food. Additionally, many were seeking a private environment with personalized care. Remaining in control of the birthing process was a priority for many of these families.

One mother, who had birthed in both environments, compared her care out of hospital and in.

Better options, more respect, better care, more information available about my birth, education was provided about my whole pregnancy, was provided with the proper information to make my own decision for myself and my baby. Overall a more personal experience. I tried going the hospital way but all I got was a number on my forehead and being called “NEXT”.

#### Empowerment

The concept of empowerment figured prominently among the women, with the desire to maintain control as a central feature of their ideal birth scenarios. Many went to great lengths to ensure their autonomy would be respected.

A sense of pride and belonging accompanied these women's goals for childbirth, and several expressed the ability to bear children physiologically as a strong identifier with womanhood.

Women have done this for millennia. I wanted to be a part of that. I wanted to experience the highest highs of giving birth but with that you know there came the lowest of—I didn't want anything to dull my experience when he was actually born. That euphoria after that. I didn't want that diminished by pain relief.

Some participants were able to overcome negative stereotypes about birth with their first child, and others only after an unsatisfying delivery. Many who did not get the empowered experiences they hoped for grieved that loss of control.

I just kinda went with the option that all my family said, which was this super highly recommended OB. But when it came down to it, I was in the delivery room and I needed to push, and they were like “You can't push yet, he's not here.” So, I had to wait 20 min, and I was crying in pain … I also felt really robbed of my experience. And it felt very unfair because I was so hopeful, you know, that he was on board with the things that I wanted.

The lack of trust between patient and provider, whether perceived or experienced, led many of the women to choose midwives for more personalized and respectful care.

#### The Midwife Relationship

Our data revealed that women regarded midwives as trustworthy and able to value a mother's goals for her childbirth. Often, respondents credited their midwives with making the birth they wanted possible. One woman discussed reaching the conclusion to see a midwife instead of an obstetrician.

I started off with an OB, and the experience was, it was interesting. When I was in there, you can tell she was already thinking about the next patient. It was my first child, too, so even though she's birthed probably millions of babies, it was my first one. She said, “Do you have any questions?” I was like, “Oh yeah, but do you want to wait until you're finished examining my pelvic area?”

Sentiments of midwives as caring, personal, and more thorough were echoed time and again. Women repeatedly mentioned the increased amount of time and discussion they had prenatally with their midwives, as well as feeling more educated about how to birth. A woman who'd had two children in birth centers explained, “It's a holistic approach to the women, and they're worried about your emotional well-being and your spiritual well-being as well as the baby. I mean, I remember my first discussions—it was questions that my OBGYN would never have asked me.”

Asked to elaborate, she continued.

They asked about sexual assault. They asked about past experiences that could affect the birth … What your relationship was like. How your partner was thinking about it … And then they explain if you had some experience, how this might impact you when you're going through contractions.

Contrary to culturally dominant beliefs that hospitals provide the safest environment, midwifery clients felt they had higher quality care and were safer birthing out of hospital.

Similarly, midwives were seen as best able to provide the sense of empowerment that women sought by centering them in their care, as compared to standard obstetric care, which was perceived to revolve around the needs of the institution and the providers.

To me it reverses the hierarchy of how it should be. Like, you should be at the top. You're the person who is giving birth. You're the center of attention for a midwife, and now you're at the bottom [in hospital]. You're the subservient person who is strapped to a table and you can't move.

### Lack of Options

As the purpose of the study was to gauge demand for a birth center attended by CNMs (ie, out-of-hospital births that could be covered by insurance), many participants expressed excitement at the prospect of such a facility, as well as disappointment with what they repeatedly described as “limited” options in El Paso.

El Paso and the surrounding area is definitely in need of a place like this. As a military wife, and covered through military insurance, the natural care options seem to be very limited, which is what worries me about having a baby here.

In sum, our data revealed that while hundreds of families in west Texas want access to midwifery care and support for physiological, patient-centered birth, they struggled to find this. While a number of our respondents obtained midwifery in an out-of-hospital setting, this was largely limited to those with the financial means.

## Discussion

### Preferences Align With Evidence

While there can be overlap between models of care and individual providers, shared decision making, freedom of movement, natural pain relief techniques, and oral nourishment during labor are key tenets of the midwifery model of care (8). We did not survey out-of-hospital birthers specifically on these questions, as in a home or birth center (which were attended by midwives among all our respondents in this category), these are standard practices and commonly cited as the reason why respondents chose to birth with midwives.

Perhaps ironically, the tenets of midwifery our respondents had difficulty accessing are supported by evidence and the medical literature. ACOG's committee opinion “Approaches to Limit Intervention During Labor and Birth,” encourages offering oral hydration, frequent position changes for mother's comfort, and nonpharmacologic pain management, as well as intermittent auscultation by a hand-held doppler. The brief also notes that one-to-one emotional support [such as that provided by CPMs] is associated with improved outcomes (2).

Likewise, the World Health Organization posits that not only do the types of practices desired by the women in our survey align with evidence for improved outcomes, but that a mother's personal satisfaction with her birth is important. Indeed, a mother's agency in the childbirth process is a human right (7).

Research specifically associates increased integration of midwives into the maternity care system with improved outcomes. A 2018 study found that greater integration was associated with more spontaneous vaginal deliveries, vaginal births after cesarean, and breastfeeding, and significantly lower rates of cesarean, preterm birth, low birth weight infants, and neonatal death (9). The findings of a 2019 comparison of midwifery and obstetric care published in *Obstetrics & Gynecology* reached similar conclusions (10).

### Human Rights in Childbirth

Many of our respondents described their hospital births in highly negative terms, such as “abusive,” “horrible,” and “terrible.” Regardless of what percentage of the childbearing population this represents, it is unacceptable. Whether women expect that childbirth should be an empowering experience or not, they deserve to be centered in their care and empowered to make their own decisions. Greater empowerment during pregnancy and birth can lead to better health outcomes for women and infants (11).

Informed consent and patient autonomy are pillars of humane healthcare and human rights. Their violation is admissible under no circumstances, and yet in the maternity care facilities of this region this appears to be flagrant and routine. Given the emotional and physical vulnerability of women during the perinatal period, it can be understood that obstetric violence might have an even more profound effect on a woman's mental health and emotional wellbeing (12). This reason alone warrants increased access to the midwifery model of care and its core practices.

We are not concluding that midwifery is the best fit or first preference for all families, only that there is a significant demand in this region that is not met. We conclude only that greater options are called for.

### Limitations

It is unsurprising that we would find a high concentration of negative impressions of hospitals in a self-selected survey of families interested in a birth center. While we know that many families profess satisfaction with their obstetrician and hospital births and that we cannot generalize our results to the larger population, our sample nonetheless demonstrates that a significant subset of the birthing population feels mistreated and even traumatized by their care.

Moreover, the population of our survey represents women who were motivated to learn about the normal process of childbirth and explore options. Families with little knowledge or experience of these possibilities may express higher satisfaction with their births simply because they do not know there is any other model. Similarly, we can suggest that if a broader array of options were available, such as midwifery care for all low-risk women, more families would express a preference for this.

### Recommendations for Future Research

A lens needs to be cast on the quality of care in El Paso. As an underserved area, provider-to-patient ratios could reveal an association between patient volume, time spent with patients through the perinatal period, interventions, and outcomes. Such an examination might also highlight how greater access to midwifery care could relieve pressure on obstetricians to see low-risk patients and allot more time for higher acuity pregnancies. As lack of insurance for midwives was cited so frequently as a barrier, ways to extend coverage to CPMs should also be explored.

## Data Availability Statement

The raw data supporting the conclusions of this article will be made available by the authors, without undue reservation.

## Ethics Statement

Ethical review and approval was not required for the study on human participants in accordance with the local legislation and institutional requirements. Written informed consent from the participants' legal guardian/next of kin was not required to participate in this study in accordance with the national legislation and the institutional requirements.

## Author Contributions

RC has designed and conducted research and wrote manuscript. RV has designed and conducted research and reviewed manuscript. CH has reviewed research design, assisted with analysis, and reviewed manuscript. CR assisted with focus groups and data analysis. All authors contributed to the article and approved the submitted version.

## Conflict of Interest

The authors declare that the research was conducted in the absence of any commercial or financial relationships that could be construed as a potential conflict of interest.

## Publisher's Note

All claims expressed in this article are solely those of the authors and do not necessarily represent those of their affiliated organizations, or those of the publisher, the editors and the reviewers. Any product that may be evaluated in this article, or claim that may be made by its manufacturer, is not guaranteed or endorsed by the publisher.
